# Effect of imeglimin on metabolic dysfunction-associated steatotic liver disease in individuals with type 2 diabetes

**DOI:** 10.1371/journal.pone.0335888

**Published:** 2025-10-31

**Authors:** Naruyoshi Gyoutoku, Yukihiro Inoguchi, Junjiro Rikitake, Mutsuyuki Demiya, Mizuki Gobaru, Ayako Nagayama, Shimpei Iwata, Nao Hasuzawa, Hitomi Nakayama, Kenji Ashida, Masatoshi Nomura

**Affiliations:** 1 Division of Endocrinology and Metabolism, Department of Internal Medicine, Kurume University School of Medicine, Kurume, Fukuoka, Japan; 2 Division of Endocrinology and Metabolism, Chikugo City Hospital, Chikugo, Fukuoka, Japan; 3 Division of Endocrinology and Metabolism, Social Insurance Tagawa Hospital, Tagawa, Fukuoka, Japan; 4 Division of Endocrinology and Metabolism, Yame General Hospital, Yame, Fukuoka, Japan; University of Diyala College of Medicine, IRAQ

## Abstract

**Objective:**

Preclinical studies, including animal and in vitro experiments, have suggested that imeglimin, a novel antidiabetic drug, may have beneficial effects on metabolic dysfunction-associated steatotic liver disease (MASLD). However, its effect on MASLD in individuals with type 2 diabetes remains unclear. This study aimed to evaluate whether imeglimin has beneficial effects in individuals with type 2 diabetes and hepatic enzyme abnormalities.

**Methods:**

This multicenter, retrospective, single-arm study included 49 individuals with type 2 diabetes who newly initiated imeglimin treatment and continued it for six months. Individuals with chronic liver diseases other than MASLD were excluded. Treatment efficacy for liver function was defined as an ALT reduction of ≥11 IU/L, corresponding to the upper interquartile range of ALT reduction. Logistic regression analysis was performed to identify predictors of this treatment efficacy.

**Results:**

Body mass index (BMI) and hemoglobin A1c (HbA1c) levels significantly decreased after six months of imeglimin treatment. Additionally, both alanine aminotransferase (ALT) and aspartate aminotransferase (AST) levels significantly decreased (ALT: 21.0 IU/L [13.0–39.0] vs. 17.0 [12.5–28.0], p* *= 0.002; AST: 21.0 IU/L [17.0–30.0] vs. 18.0 [16.0–26.0], p* = *0.010, respectively). Logistic regression analysis showed that baseline ALT and AST levels were significant predictors of treatment efficacy for liver function, after adjusting for HbA1c and BMI levels (OR 1.06, 95% CI 1.02–1.12, p = 0.006; OR 1.12, 95% CI 1.06–1.23, p = 0.002, respectively). Furthermore, the receiver operating characteristic (ROC) curve for baseline ALT as a predictor demonstrated excellent discriminatory performance (Area under the curve: 0.905, p* *= 0.004). A cutoff value of 25 IU/L yielded 100% sensitivity and 77.8% specificity.

**Conclusion:**

This exploratory study suggests that imeglimin may have beneficial effects in individuals with type 2 diabetes and elevated ALT (≥25 IU/L), potentially associated with MASLD. Randomized controlled trials are needed to confirm these findings.

## Introduction

Metabolic dysfunction-associated steatotic liver disease (MASLD), formerly referred to as non-alcoholic fatty liver disease, is a growing global health concern [1]. MASLD is characterized by hepatic steatosis accompanied by at least one cardiometabolic risk factor, such as an elevated body mass index (BMI), increased waist circumference, impaired glucose tolerance, dyslipidemia, or hypertension [2]. Metabolic dysfunction-associated steatohepatitis (MASH) represents the inflammatory and fibrotic spectrum of MASLD and signifies a more severe progression of the underlying metabolic liver disease [1]. MASH is recognized as an independent risk factor for cardiovascular disease [1,3] and is increasingly acknowledged as a leading cause of cirrhosis and hepatocellular carcinoma (HCC) in the general population [1,4]. The *Standards of Care in Diabetes—2025,* issued by the American Diabetes Association (ADA) recommend appropriate screening for MASLD in patients with type 2 diabetes to prevent future cirrhosis, HCC, liver transplantation, and all-cause mortality [5]

Lifestyle interventions, such as dietary modifications and exercise, have been shown to improve MASLD [5,6]. Additionally, several pharmacological agents, including vitamin E, sodium-glucose cotransporter 2 (SGLT2) inhibitors, glucagon-like peptide-1 receptor agonists (GLP-1RAs), and pioglitazone, have demonstrated efficacy in MASLD, and their use is supported by clinical evidence [5,6]. Nevertheless, given the multifactorial pathogenesis of MASLD, treatment responses are heterogeneous and many patients do not achieve sufficient improvement, indicating a continuing unmet need for additional therapeutic options.

Imeglimin is a novel antidiabetic agent with a unique chemical structure that bears some resemblance to metformin. Preclinical and clinical studies have demonstrated that imeglimin improves both insulin resistance and insulin secretion. In addition, imeglimin has been reported to have a lower risk of lactic acidosis [7–11]. These features suggest that imeglimin exerts its antidiabetic effects through novel mechanisms of action, although these mechanisms have not yet been fully elucidated.

Beyond improving glycemic control, several animal studies have indicated that imeglimin may reduce hepatic fat accumulation in obese mice fed a high-fat, high-sucrose diet [12,13]. Furthermore, imeglimin has been shown to attenuate inflammation and oxidative stress and to prevent hepatic fibrosis in non-diabetic mice with MASH induced by a choline-deficient, high-fat diet [14]. These findings raise the possibility that imeglimin could serve as a novel therapeutic option for MASLD in patients with type 2 diabetes. However, clinical evidence regarding the effects of imeglimin on liver function remains limited. This study aims to evaluate whether imeglimin has beneficial effects in individuals with type 2 diabetes and elevated liver enzyme levels.

## Materials and methods

### Participants

We conducted a multicenter, retrospective, single-arm observational study to examine the effect of imeglimin treatment on liver function in a real-world clinical setting among individuals with type 2 diabetes. A total of 49 individuals with type 2 diabetes receiving medical care at Kurume University Hospital, Social Insurance Tagawa Hospital, Yame General Hospital, and Chikugo City Hospital were recruited from December 2021 to April 2024. All participants were newly treated with imeglimin and continued treatment for six months. In accordance with recent consensus statements [15,16], MASLD is defined as hepatic steatosis in the presence of cardiometabolic risk factors such as type 2 diabetes. Because hepatic steatosis could not be confirmed by imaging or histology in this study, we defined MASLD clinically as the presence of type 2 diabetes with elevated ALT (≥25 IU/L), alcohol consumption below the threshold for alcohol-related liver disease (<women: 350 mg/week, men: 420 mg/week), and the absence of other chronic liver diseases. Patients with viral hepatitis, autoimmune hepatitis, drug-induced liver injury, or primary biliary cholangitis were excluded based on medical history, laboratory data available in clinical records, and physician judgment. However, systematic serological testing for viral and autoimmune markers was not performed in all participants. All procedures were conducted in accordance with the ethical principles outlined in the Declaration of Helsinki. This multi-center study was conducted under a single centralized approval granted by the Clinical Research Ethics Committee of Kurume University Hospital (approval no. 22034). All participating institutions relied on this central approval in accordance with their institutional policies; therefore, separate local approvals were not required. This study utilized data from the “Development and Analysis of a Diabetes Registry in Chikugo”, and eligible participants were identified by reviewing their medical records. Data were accessed for research purposes between May 2024 and December 2024. The authors had access to identifiable information during data collection; however, all data were anonymized prior to analysis.

### Data collection

All data were retrospectively collected from medical records. Hemoglobin A1c (HbA1c) levels were measured using a standard high-performance liquid chromatography method and reported according to the National Glycohemoglobin Standardization Program. The Fibrosis-4 (Fib-4) index was calculated using the following formula: age (years) × aspartate aminotransferase (AST) (U/L)/ [platelet count (10⁹/L) × √alanine aminotransferase (ALT) (U/L)]. Hypertension was defined as a systolic blood pressure >140 mmHg, diastolic blood pressure >90 mmHg, or the current use of any antihypertensive medication. Hyperlipidemia was defined as a serum concentration of low-density lipoprotein cholesterol (LDL-C) ≥120 mg/dL and triglycerides ≥150 mg/dL, in accordance with Japan Atherosclerosis Society criteria, or the current use of lipid-lowering agents. Macrovascular disease was defined as a history of acute myocardial infarction, angina pectoris confirmed by clinically significant obstruction on coronary angiography, revascularization via angioplasty or coronary artery bypass surgery, cerebral infarction, and peripheral vascular disease.

### Statistical analyses

Continuous variables are presented as mean ± standard deviation (SD) or median (25th-75th percentile), while categorical variables are presented as numbers and percentages. Differences between groups were analyzed using the unpaired *t*-test or the Wilcoxon signed-rank test for continuous variables and the chi-squared test for categorical variables. Changes in concomitant medication use between baseline and study completion were evaluated using Bowker’s test for paired categorical data. Logistic regression analysis was performed to identify predictors of treatment efficacy, aiming to determine which populations benefit most from the treatment. Treatment efficacy for liver function was defined as an ALT reduction in the upper interquartile range (corresponding to an ALT reduction of ≥11 IU/L, S1 Fig). Statistical analyses were performed using JMP version 18.2.1 (SAS Institute Inc., Cary, NC, USA). A two-sided *p*-value < 0.05 was considered statistically significant.

## Results

The characteristics of the 49 participants are summarized in Table 1. After six months of imeglimin treatment, significant reductions were observed in body weight, body mass index (BMI), and HbA1c levels (64.7 ± 17.0 kg vs. 63.7 ± 16.6 kg, p* *= 0.034; 24.8 ± 5.2 kg/m^2^ vs. 24.4 ± 5.0 kg/m^2^, p* *= 0.033, 8.2% [7.6–9.1] vs. 7.4% [7.0–7.9], p* *< 0.0001). Serum ALT and AST levels also significantly decreased (ALT: 21.0 IU/L [13.0–39.0] vs. 17.0 IU/L [12.5–28.0], p* *= 0.002; AST: 21.0 IU/L [17.0–30.0] vs. 18.0 IU/L [16.0–26.0], p* *= 0.010). During the observation period, three adverse events were observed. All were gastrointestinal symptoms, which required dose reduction but did not lead to treatment discontinuation.

**Table 1:** Clinical characteristics at baseline and at the end of the study.

Variable	Before	After	P-value
Age, yeas old	65.2 ± 14.3	–	–
Sex (Female), n (%)	15 (30.6)	–	–
Body weight, Kg	64.7 ± 17.0	63.7 ± 16.6	0.034^a^
BMI, kg/m^2^	24.8 ± 5.2	24.4 ± 5.0	0.033^a^
HbA1c, %	8.2 [7.6–9.1]	7.4 [7.0–7.9]	<0.0001^b^
AST, IU/L	21.0 [17.0–30.0]	18.0 [16.0–26.0]	0.010^b^
ALT, IU/L	21.0 [13.0–39.0]	17.0 [12.5–28.0]	0.002^b^
Fib4 index	1.3 [0.9–1.7]	1.4 [0.9–1.7]	0.224^b^
LDL-C, mg/dL	97.0 [82.8–116.3]	101.0 [83.8–122.3]	0.711^b^
HDL-C, mg/dL	50.0 [40.8–63.0]	48.0 [41.0–60.0]	0.478^b^
Triglyceride, mg/dL	115.0 [82.0–200.0]	119.0 [81.5–167.0]	0.649^b^
Platelet count, × 10⁴/mm³	23.1 [19.1-27.9]	23.1 [20.1-29.5]	0.367^b^
Hypertension, n (%)	27 (55.1)	–	–
Dyslipidemia, n (%)	30 (61.2)	–	–
Macrovascular diseases, n (%)	7 (14.3)	–	–
DPP4 inhibitor use, n (%)	23 (46.9)	23 (46.9)	1.000^c^
SGLT2 inhibitor use, n (%)	30 (61.2)	31 (63.3)	0.564^c^
Metformin use, n (%)	26 (53.1)	27 (55.1)	1.000^c^
Sulfonylurea use, n (%)	16 (32.7)	17 (34.7)	0.317^c^
GLP-1RA use, n (%)	18 (36.7)	16 (32.7)	0.317^c^
GLP-1/GIPRA use, n (%)	0 (0.0)	3 (0.06)	–
Thiazolidine use, n (%)	1 (2.0)	1 (0.02)	1.000^c^
Insulin use, n (%)	18 (36.7)	17 (34.7)	0.317^c^
ACE inhibitor/ARB use, n (%)	20 (40.8)	20 (40.8)	1.000^c^
Statin use, n (%)	21 (42.9)	21 (42.9)	1.000^c^
Fibrate use, n (%)	3 (6.1)	3 (6.1)	1.000^c^
Pemafibrate use, n (%)	3 (6.1)	3 (6.1)	1.000^c^
Antiplatelet use, n (%)	7 (14.3)	7 (14.3)	1.000^c^

^a^Caluculated using t-test

^b^Caluculated using Wilcoxon signed-rank test

^c^Caluculated using Bowker’s test

Missing number of patients: LDL-C n = 11, HDL-C n = 7, Triglyceride n = 4, Fib-4 index n = 1, Platelet count n = 1

Abbreviation: BMI, body mass index; HbA1c, hemoglobin A1c; AST, aspartate aminotransferase; ALT, alanine aminotransferase; LDL-C, low density lipoprotein cholesterol; HDL-C, high density lipoprotein cholesterol; DPP4, dipeptidyl peptidase-4; SGLT2, sodium glucose co transporter 2; GLP1-RA, glucagon-like peptide-1 receptor agonists; GLP-1/GIPRA, glucagon-like peptide-1/ glucose-dependent insulinotropic polypeptide receptor co agonists; ACE, angiotensin converting enzyme; ARB, angiotensin II receptor blocker

**Fig 1:**
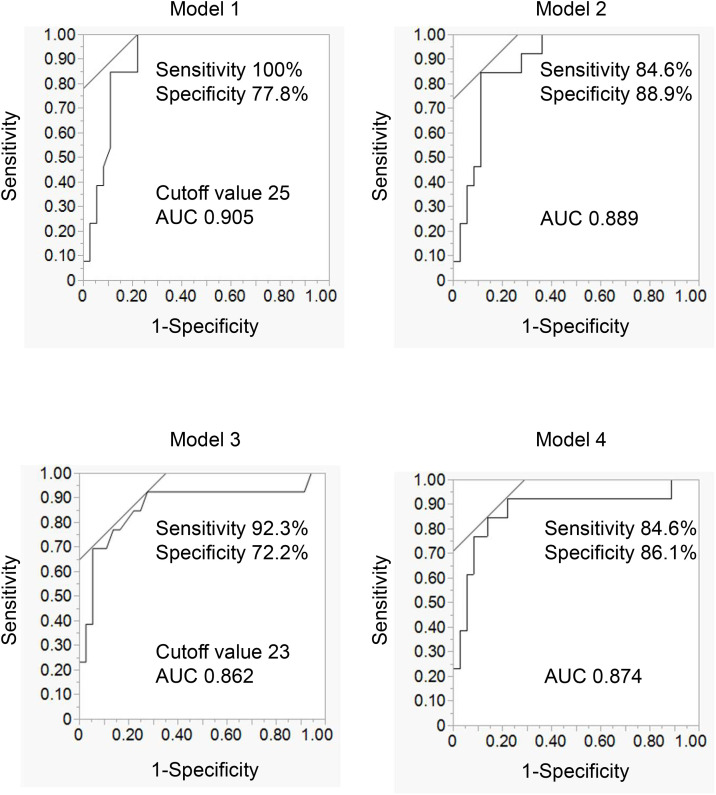
AUC of various models to predict imeglimin efficacy for liver function evaluated by ROC curves. ROC curves demonstrate the relative ability of ALT alone (Model 1), ALT, BMI and HbA1c (Model 2) **(A)**, AST alone (Model 3), and AST, BMI and HbA1c (Model 4). AUC, area under the curve. ROC, receiver operating characteristic.

Liver function improvement was defined as a reduction in ALT ≥ 11 IU/L, corresponding to the upper interquartile range of ALT reduction (S1 Fig). In univariate analysis, baseline ALT and AST were the only variables significantly different between the efficacy and non-efficacy group (ALT: 42.0 IU/L [36.0–65.5] vs 15.5 IU/L [12.0–22.8], *p* < 0.0001; AST: 38.0 IU/L [27.0–56.0] vs 20.0 IU/L [17.0–23.8], p = 0.0001) (Table 2). Logistic regression analysis demonstrated that baseline ALT and AST levels remained significantly associated with treatment-related improvements in liver enzymes, even after adjustment for HbA1c and BMI. (ALT: Odd ratio [OR] 1.06, 95% Confidence Interval [CI] 1.02–1.12, p = 0.006; AST: OR 1.12, 95%CI 1.06–1.23, p = 0.002) (Table 3). Receiver operating characteristic (ROC) curve analysis demonstrated excellent predictive performance of baseline ALT and AST levels for treatment efficacy, with area under the curve (AUCs) of 0.905 and 0.862, respectively. The optimal cutoff values were 25 IU/L for ALT and 23 IU/L for AST (Fig 1). Since ALT yielded a higher AUC, it may be a more reliable predictor of hepatic improvement, with the cutoff value of 25 IU/L providing 100% sensitivity and 77.8% specificity.

**Table 2:** Comparison in baseline variables between efficacy group and non-efficacy group.

Variables	Efficacy+ (n = 13)	Non-efficacy – (n = 36)	p-value
Age, years old	60.8 ± 14.9	66.8 ± 13.9	0.196^a^
Sex (Female), n (%)	4 (30.8)	11 (30.6)	0.989^c^
Body weight, kg	70.2 ± 21.5	62.8 ± 14.9	0.182^a^
BMI, kg/m^2^	25.8 ± 7.3	24.5 ± 4.2	0.453^a^
HbA1c, %	8.6 [7.7-10.1]	8.2 [7.5-8.8]	0.267^b^
AST, IU/L	38.0 [27.0-56.0]	20.0 [17.0-23.8]	0.0001^b^
ALT, IU/L	42.0 [36.0-65.5]	15.5 [12.0-22.8]	<0.0001^b^
Fib4 index	1.7 [1.0-2.6]	1.2 [0.9-1.7]	0.065^b^
LDL-C, mg/dL	84.0 [72.0-135.0]	100.0 [87.0-115.0]	0.471^b^
HDL-C, mg/dL	44.0 [36.0-54.0]	51.0 [43.0-64.0]	0.064^b^
TG, mg/dL	121.0 [97.3-233.0]	108.0 [78.0-185.5]	0.323^b^
Platelet count, × 10⁴/mm³	19.3 [16.1-27.4]	23.3 [20.1-27.9]	0.167^b^
Hypertension, n (%)	9 (69.2)	18 (50.0)	0.227^c^
Dyslipidemia, n (%)	8 (61.5)	22 (61.1)	0.978^c^
Macrovascular diseases, n (%)	2 (15.4)	5 (13.9)	0.896^c^
DPP4 inhibitor use, n (%)	4 (30.8)	19 (52.8)	0.168^c^
SGLT2 inhibitor use, n (%)	6 (46.2)	24 (66.7)	0.197^c^
Metformin use, n (%)	8 (61.5)	18 (50)	0.473^c^
Sulfonylurea use, n (%)	5 (38.5)	11 (30.6)	0.605^c^
GLP-1RA use, n (%)	5 (38.5)	13 (36.1)	0.881^c^
Thiazolidine use, n (%)	1 (7.7)	0 (0)	0.100^c^
Insulin use, n (%)	4 (30.8)	14 (38.9)	0.600^c^
ACE inhibitor/ARB use, n (%)	7 (53.9)	13 (36.1)	0.268^c^
Statin use, n (%)	4 (30.8)	17 (47.2)	0.298^c^
Fibrate use, n (%)	1 (7.7)	2 (5.6)	0.788^c^
Pemafibrate use, n (%)	2 (15.4)	1 (2.8)	0.132^c^
Antiplatelet use, n (%)	2 (15.4)	5 (13.9)	0.896^c^

^a^Caluculated using t-test

^b^Caluculated using Wilcoxon signed-rank test

^c^Caluculated using chi-squared test

Missing number of patients: Efficacy group: LDL-C n = 4, HDL-C n = 2, Triglyceride n = 1; Non-efficacy group: LDL-C n = 7, HDL-C n = 5, Triglyceride n = 3, Fib-4 index n = 1, Platelet count n = 1

Abbreviation: BMI, body mass index; HbA1c, hemoglobin A1c; AST, aspartate aminotransferase; ALT, alanine aminotransferase; LDL-C, low density lipoprotein cholesterol; HDL-C, high density lipoprotein cholesterol; DPP4, dipeptidyl peptidase-4; SGLT2, sodium glucose co transporter 2; GLP-1RA, glucagon-like peptide-1 receptor agonists; ACE, angiotensin converting enzyme; ARB, angiotensin II receptor blocker

**Table 3:** Logistic regression analysis to predict the efficacy for liver function.

ALT						
	Model 1			Model 2		
Variables	Odd ratio	95% CI	P-value	Odd ratio	95% CI	P-value
ALT, per IU/L	1.06	1.03-1.12	0.004	1.06	1.02-1.12	0.006
BMI, per kg/m^2^	–	–	–	1.02	0.89-1.16	0.776
HbA1c, per %	–	–	–	1.08	0.63-1.67	0.754
AST						
	Model 3			Model 4		
Variables	Odd ratio	95% CI	P-value	Odd ratio	95% CI	P-value
AST, per IU/L	1.12	1.06-1.23	<0.0001	1.12	1.06-1.23	0.002
BMI, per kg/m^2^	–	–	–	0.98	0.84-1.14	0.801
HbA1c, per %	–	–	–	1.18	0.69-1.86	0.478

Data are presented as OR and 95% CI. OR represents the increase in odds of efficacy on the liver function for every unit increase in the dependent continuous variable. Model 1: ALT alone. Model 2: ALT, BMI and HbA1c. Model 3: AST alone, Model 4: AST, BMI and HbA1c.

Abbreviation: BMI, body mass index; HbA1c, hemoglobin A1c; AST, aspartate aminotransferase; ALT, alanine aminotransferase

**Fig 2:**
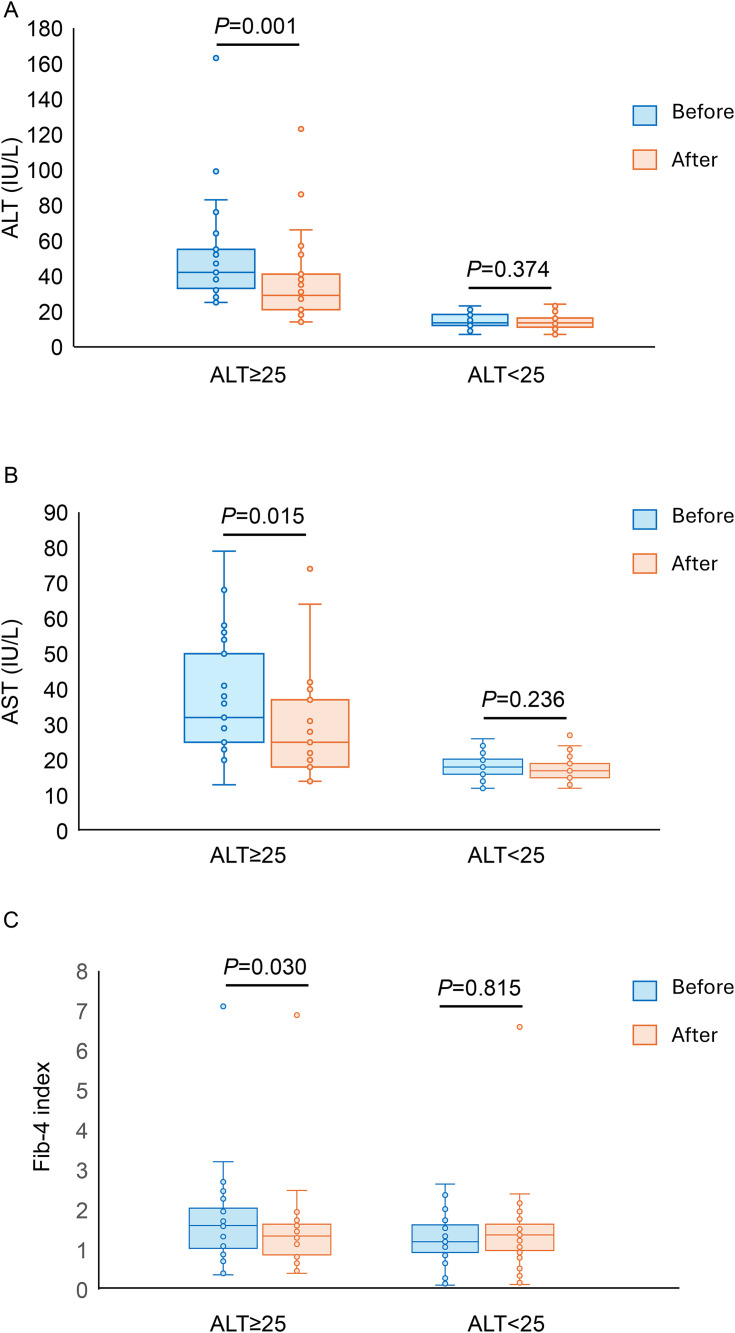
Comparison of ALT (A), AST (B), and Fib-4 index (C) before and after imeglimin treatment in ALT ≥ 25 IU/L and ALT < 25IU/L groups. N = 21 for ALT ≥ 25 IU/L group, n = 28 for ALT < 25IU/L group. Data are presented as median (IQR). Wilcoxon signed-rank sum test was used to compare ALT, AST, and Fib-4 index before and after imeglimin treatment. Missing number of patients: ALT < 25IU/L group: Fib-4 index n = 1.

In participants with baseline ALT ≥ 25 IU/L, both ALT and AST levels significantly decreased after treatment (ALT: 42.0 IU/L [32.5–59.5] vs. 29.0 IU-L [19.5–46.5], p* *= 0.001; AST: 32.0 IU/L [25.0–52.0] vs. 25.0 IU/L [18.0–38.5], p* *= 0.015). In contrast, no significant changes were observed in the ALT < 25 IU/L group (ALT: 13.5 IU/L [12.0–18.8] vs. 13.5 IU/L [11.0–16.8], p* *= 0.374; AST: 18.0 IU/L [16.0–20.8] vs. 17.0 IU/L [15.0–19.0], p* *= 0.236) (Fig 2A, 2B). Moreover, the Fib-4 index was significantly decreased in the ALT ≥ 25 IU/L group (1.6 [1.0–2.1] vs. 1.4 [0.9–1.7], p* *= 0.030), but not in the ALT < 25 IU/L group (1.2 [0.9–1.6] vs. 1.4 [0.9–1.6], p* *= 0.815) (Fig 2C).

Finally, the influence of concomitant glucose-lowering therapies was investigated in the ALT ≥ 25 IU/L group. At baseline, 52.3% were treated with SGLT2 inhibitors and 38.1% were using GLP-1 receptor agonists (GLP-1RAs); only one participant was using pioglitazone. To isolate the effect of imeglimin, participants who initiated or discontinued SGLT2 inhibitors, GLP-1RAs and dual GLP1/GIP RAs during the study period were excluded, resulting 9 participants in the SGLT2 inhibitor non-user group, 8 in the SGLT2 inhibitor user group, 11 in the GLP-1RAs non-user group, and 6 in the GLP-1RAs user group. Significant reductions in ALT, AST and the Fib-4 index were observed in both non-user groups (S2A – S2F Fig). In contrast, no significant reductions were observed in the GLP-1RA user groups, whereas a significant reduction in ALT was observed in the SGLT2 inhibitor user group. (S2A, S2D- S2F Fig).

## Discussion

Preclinical studies, including animal and in-vitro experiments, have suggested that imeglimin may exert beneficial effects on MASLD [12–14]. However, clinical evidence showing its effects on MASLD in individuals with type 2 diabetes remains limited. In the present study, we demonstrated that the efficacy of imeglimin in improving liver function was significantly associated with baseline AST and ALT levels. ROC curve analysis identified a baseline ALT cutoff value of 25 IU/L as an excellent predictor of treatment response. Imeglimin significantly reduced both ALT and AST levels in individuals with ALT ≥ 25 IU/L, potentially indicative of MASLD, while no significant reduction was observed in those with ALT < 25 IU/L. Furthermore, a significantly reduction in the Fib-4 index was observed only in the ALT ≥ 25 IU/L group.

According to the American Association for the Study of Liver Diseases (AASLD), ALT levels exceeding 30 IU/L may suggest the presence of nonalcoholic fatty liver disease [17]. In a biopsy-based study, Castera et al. demonstrated that men with ALT ≥ 30 IU/L and women with ALT ≥ 20 IU/L had a higher prevalence of nonalcoholic steatohepatitis and liver fibrosis [18]. Based on these findings, it is plausible that the ALT ≥ 25 IU/L group in our study included individuals with hepatic steatosis, inflammation, or fibrosis, in whom imeglimin exerted beneficial effects.

The pathogenesis of MASLD and MASH is complex and multifaceted. Only 5–10% of MASLD patients progress to MASH, while the majority (90–95%) present with simple steatosis, commonly referred to as fatty liver [19]. The widely accepted multiple-hit hypothesis implicates insulin resistance as the central mechanism, promoting hepatic de novo lipogenesis (DNL) and increasing circulating free fatty acids (FFA) via enhanced adipose tissue lipolysis [20]. These FFAs are taken up by the liver, contributing to triglyceride accumulation, mitochondrial dysfunction, and the generation of reactive oxygen species (ROS) and endoplasmic reticulum (ER) stress, which further drive hepatic inflammation and injury [21].

Imeglimin has demonstrated multifaced hepatic effects in preclinical models. It ameliorated hepatic steatosis by enhancing fatty acid oxidation, stimulating glucagon secretion, and suppressing DNL, while also improving mitochondrial function [12,13]. Specifically, imeglimin partially inhibits mitochondrial Complex I and restores Complex III activity in hepatocytes, resulting in reduced ROS production and increased ATP levels [12]. These mitochondrial effects may contribute to the observed hepatoprotection, as ROS overproduction and ATP depletion are associated with hepatocyte injury [22]. Kaji et al. reported that imeglimin attenuated steatosis, inflammation, and fibrosis in a non-diabetic MASH mouse model induced by a choline-deficient, high-fat diet (CDA-HFD) [14]. Imeglimin increased hepatic expression of fatty acid oxidation markers, such as peroxisome proliferator-activated receptor-α (PPAR-α), carnitine palmitoyltransferase I (CPT-1), and acyl-CoA oxidase 1 (ACOX1), and decreased hepatic oxidative stress markers, including 4-hydroxynonenal (4-HNE) and malondialdehyde (MDA). It also suppressed hepatic macrophage infiltration and proinflammatory cytokines, such as tumor necrosis factor-α (TNF-α) and interleukin-6 (IL-6), while reducing fibrosis markers, including collagen type 1, α-smooth muscle actin (α-SMA), and tissue inhibitor of metalloproteinase-1 (TIMP-1), and galectin-3 expression. These findings suggest that imeglimin may directly attenuate hepatic steatosis, inflammation, and fibrosis.

In addition to these preclinical observations, recent clinical studies have also reported hepatic benefits of imeglimin in patients with type 2 diabetes. Uto et al. [23] demonstrated improvements in liver enzyme levels and elastography-based indices after six months of imeglimin therapy. Tanaka et al. [24] observed reductions in noninvasive fibrosis markers, including Fib-4 index and aspartate aminotransferase to platelet ratio index (APRI), particularly among patients at higher baseline fibrosis risk. Furthermore, Fukunaga et al. [25] showed that imeglimin improved ALT, AST, and fibrosis-related indices such as FIB-4 and FAST score, although no significant changes were detected in liver fat content or stiffness. These findings are consistent with the present results, while our study extends prior work by demonstrating that improvements in ALT, AST and FIB-4 were confined to patients with elevated baseline ALT (≥25 IU/L), thereby highlighting the potential clinical utility of ALT as a simple marker for stratifying treatment response.

In the present study, the observed reduction in ALT in the ALT ≥ 25 IU/L group may reflect improvements in hepatic steatosis and inflammation, as suggested by both preclinical studies and recent clinical trials reporting decreases in Fib-4 following imeglimin therapy [24,25]. The Fib-4 index levels in this group decreased from 1.6 [1.0–2.1] to 1.4 [0.9–1.7], slightly above the normal range [5,26]. Although the Fib-4 index is useful noninvasive marker of liver fibrosis, its diagnostic performance is reportedly reduced in individuals with type 2 diabetes [27,28]. Thus, while our findings hint at a potential antifibrotic effect of imeglimin, further investigation is warranted to substantiate this.

With regard to combination therapy, trends toward reduced ALT and AST levels were observed in individuals concurrently using SGLT2 inhibitors or GLP-1 receptor agonists. However, a statistically significant reduction was observed only in ALT levels among those using SGLT2 inhibitors. This limited significance may be due to the small sample size and does not exclude a possible synergistic effect, particularly in patients receiving GLP-1 receptor agonists. The effect of imeglimin was also observed in the subgroup using SGLT2 inhibitors. While both agents have been associated with enhanced fatty acid oxidation, the underlying mechanisms appear to differ: imeglimin improves mitochondrial function and thereby facilitates more efficient fatty acid oxidation at the cellular level [12], whereas SGLT2 inhibitors induce a systemic substrate shift from glucose toward fatty acid and ketone body utilization through glucosuria-induced energy loss [29]. In addition, preclinical studies have suggested that both agents may attenuate hepatic inflammation and fibrosis through mitochondrial protection, oxidative stress reduction, or metabolic modulation [12,14,29], although the extent to which these effects overlap remains uncertain. Taken together, these findings suggest that the hepatic benefits of imeglimin under concomitant SGLT2 inhibition are mediated by complementary and at least partly independent mechanisms. Further studies with larger cohorts are warranted to clarify these interactions more definitively.

This study has several limitations. First, it was conducted as a one-arm, uncontrolled study, limiting our ability to ascribe causality to imeglimin. Second, although we used serum ALT as a representative marker of liver function to stratify patients, we recognize that ALT is only a surrogate marker and does not directly reflect hepatic steatosis, inflammation, or fibrosis. Objective assessment using imaging modalities such as MRI or ultrasonography would be required to confirm hepatic fat reduction. However, because this was a retrospective, real-world, multicenter analysis, standardized imaging data were unavailable. Therefore, our findings should be interpreted as statistical associations based on biochemical surrogates rather than direct evidence of histological or imaging-based improvement. In addition, the threshold of ALT reduction (≥11 IU/L) used to stratify treatment response was derived from the statistical distribution (upper interquartile range) rather than from an established clinical cutoff, and should be regarded as an exploratory criterion. Third, the small sample size and short duration reduce the generalizability of our findings. In addition, the possibility of regression to the mean cannot be completely excluded, as patients with higher baseline ALT levels are more likely to show a reduction. Nevertheless, elevated baseline values do not necessarily normalize spontaneously, and the significant decrease observed only in patients with high ALT suggests a clinically meaningful therapeutic effect of imeglimin. Fourth, although patients with viral hepatitis, autoimmune hepatitis, drug-induced liver injury, and other chronic liver diseases were excluded based on medical history and available clinical data, systematic serological testing for viral and autoimmune markers was not performed in all participants. Moreover, HCC was not systematically ruled out in this cohort; although no participants had clinical or imaging findings suggestive of HCC in their records, the possibility of unrecognized subclinical liver disease, including HCC, cannot be completely excluded.

## Conclusion

This exploratory study suggests that imeglimin may improve liver enzyme levels and the Fib-4 index in individuals with type 2 diabetes who exhibit elevated ALT levels (≥ 25 IU/L), potentially indicating coexisting MASLD. These findings support the need for large-scale, randomized controlled trials to confirm the hepatic benefits of imeglimin and to clarify its role in MASLD treatment.

## Supporting information

S1 Fig Change in ALT before and after treatment. (PDF)

S2 Fig Changes in liver enzymes with combination therapy. (PDF)

S3 Data Raw dataset of characteristics. (PDF)
