# Supplementary material for: Effect of imeglimin on metabolic dysfunction-associated steatotic liver disease in individuals with type 2 diabetes
Source: PLoS One. 2025 Oct 31;20(10):e0335888. doi: 10.1371/journal.pone.0335888 (PMC12578173; doi:10.1371/journal.pone.0335888)
Supplement: S1 Fig — (PDF) [file pone.0335888.s001.pdf]

S1 Figure

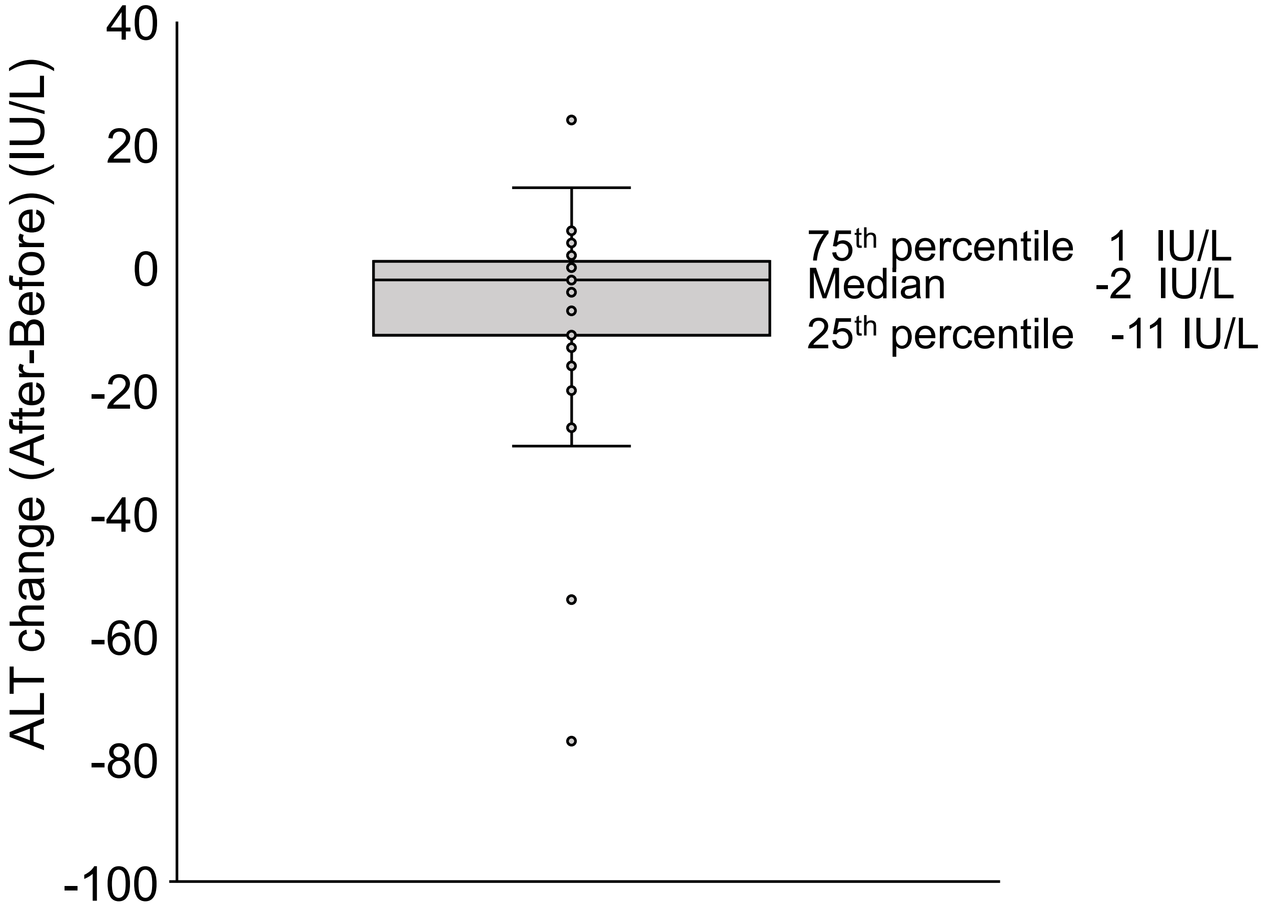

Change in ALT before and after treatment, presented as quartiles. The 75th percentile was 1 IU/L, the median was -2 IU/L, and the 25th percentile was -11 IU/L.
