# Supplementary material for: Effect of imeglimin on metabolic dysfunction-associated steatotic liver disease in individuals with type 2 diabetes
Source: PLoS One. 2025 Oct 31;20(10):e0335888. doi: 10.1371/journal.pone.0335888 (PMC12578173; doi:10.1371/journal.pone.0335888)
Supplement: S2 Fig — (PDF) [file pone.0335888.s002.pdf]

S2 Figure

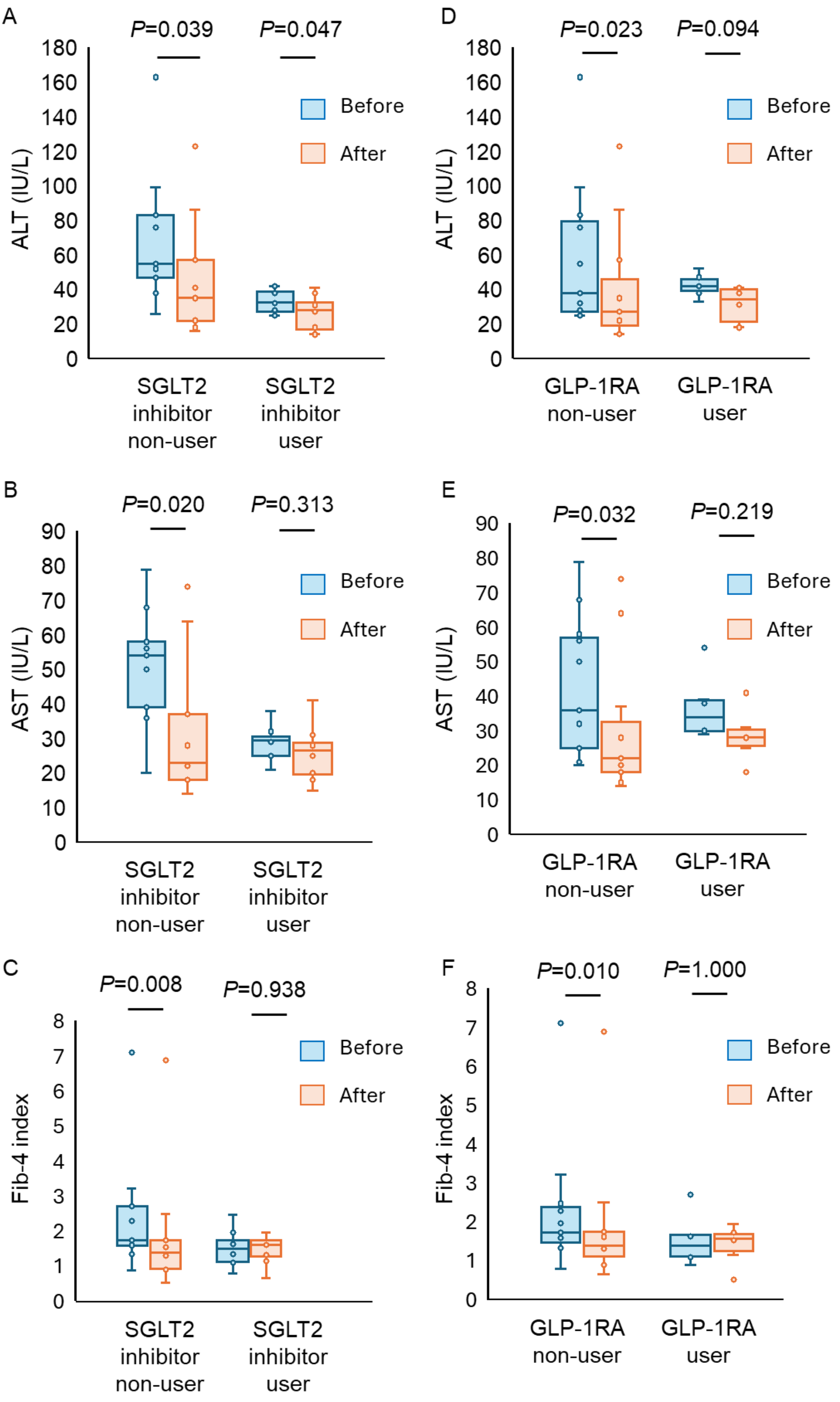

Comparison of ALT (A, D), AST (B, E), and Fib-4 index (C, F) before and after imeglimin treatment in SGLT2 Inhibitor and GLP-1RA user and non-user groups in ALT ≥25IU/L group. N=8 for SGLT2 inhibitor user group and n = 9 for non-user group. N = 6 for GLP-1RA user group and n = 11 for non-user group. Data are presented as median (IQR). Wilcoxon signed-rank sum test was used to compare ALT before and after imeglimin treatment.
