# Supplementary material for: Effect of imeglimin on metabolic dysfunction-associated steatotic liver disease in individuals with type 2 diabetes
Source: PLoS One. 2025 Oct 31;20(10):e0335888. doi: 10.1371/journal.pone.0335888 (PMC12578173; doi:10.1371/journal.pone.0335888)
Supplement: S3 Data — (PDF) [file pone.0335888.s003.pdf]

Patient No.	Sex Female=0	Age
1	0	71
2	0	83
3	0	72
4	0	44
5	0	27
6	0	73
7	0	80
8	0	71
9	0	65
10	1	42
11	1	71
12	1	64
13	1	58
14	1	60
15	1	78
16	1	62
17	1	85
18	1	76
19	1	58
20	1	71
21	1	75
22	1	67
23	1	56
24	1	83
25	1	79
26	1	76
27	1	85
28	1	64
29	0	85
30	0	74
31	0	53
32	0	76
33	0	80
34	0	37

35	1	67
36	1	56
37	1	67
38	1	55
39	1	34
40	1	44
41	1	70
42	1	57
43	1	49
44	1	54
45	1	46
46	1	77
47	1	68
48	1	73
49	1	76

BMI before	BMI after	HbA1c before
28.5	30.7	8.6
22.0	22.2	8.8
26.6	26.6	7.6
27.7	27.3	7.5
25.1	27.4	9.6
21.7	18.8	7.5
18.2	18.4	7.3
22.7	23.3	10.3
24.9	23.6	8.2
21.0	22.1	8.9
22.7	22.0	6.6
24.4	24.4	7.6
24.7	24.1	7.0
32.9	32.1	6.6
23.6	24.3	7.1
20.9	21.1	7.2
20.9	19.7	7.8
20.1	20.4	8.8
25.2	23.2	7.9
28.7	30.1	6.4
21.9	21.8	15.6
26.3	26.6	9.2
22.4	22.5	8.0
22.3	21.7	7.6
25.7	23.9	8.6
23.6	23.5	8.6
20.3	19.1	8.1
28.5	28.5	9.2
21.0	20.6	7.7
21.6	20.7	7.7
28.6	28.0	9.2
37.1	32.6	7.0
15.0	13.6	8.7
33.7	33.1	9.4

23.6	23.9	6.8
21.8	23.2	10.7
23.9	24.3	9.6
31.3	31.8	11.4
19.4	19.7	11.0
25.9	24.7	8.3
20.7	17.4	7.8
26.3	25.0	8.6
36.4	35.4	8.4
27.4	28.3	7.2
41.0	38.0	10.6
20.5	19.2	7.7
22.3	22.4	8.4
28.7	27.6	8.5
16.8	17.2	7.7

HbA1c after	HDL-C before	HDL-C after
9.3	63	56
8.1	52	47
7.4	77	69
6.5	36	38
7.9	31	54
6.7	29	31
7.9	ND	ND
7.5	51	36
7.1	67	48
7.7	ND	ND
6.6	ND	ND
7.3	48	50
6.9	42	43
6.5	69	60
7.1	ND	ND
7.6	50	46
7.4	50	35
7.3	56	53
7.2	40	51
6.0	36	43
5.9	ND	ND
7.8	48	51
7.3	64	61
7.4	72	60
7.4	67	49
7.1	61	61
6.5	63	55
7.7	48	43
7.5	51	60
6.8	63	66
7.8	ND	ND
6.9	64	74
8.5	87	95
10.4	36	48

6.5	36	38
6.3	44	48
8.3	36	38
9.4	44	56
9.7	48	42
7.8	58	63
9.9	ND	ND
9.1	32	32
7.3	41	33
7.7	66	71
9.3	36	37
7.0	54	47
7.7	52	43
7.6	43	44
7.8	45	38

LDL-C before	LDL-C after	Triglyceride before
87	84	165
114	142	116
94	101	82
130	121	211
209	178	271
79	98	67
ND	ND	ND
98	102	175
103	71	95
100	92	70
ND	ND	ND
95	102	89
87	85	160
109	120	92
ND	ND	ND
113	101	65
87	65	136
109	106	108
155	143	405
104	134	159
170	140	337
ND	ND	230
ND	ND	51
62	97	45
87	98	90
92	83	82
ND	ND	107
ND	ND	74
60	63	122
78	106	55
137	127	182
93	70	113
117	126	59
ND	ND	358

84	89	92
216	131	115
67	79	233
49	41	53
51	47	341
96	156	204
ND	ND	ND
133	107	233
125	112	413
105	110	196
ND	ND	116
ND	ND	126
116	91	109
65	74	91
77	90	48

Triglyceride after	AST before	AST after
328	24	19
142	26	22
119	17	17
127	15	12
151	16	17
124	12	17
ND	17	13
377	12	13
87	13	14
75	20	18
ND	20	24
81	19	21
170	18	15
80	16	14
ND	22	23
61	18	17
112	17	15
97	21	19
378	21	19
126	20	27
186	20	17
314	25	27
110	17	14
53	19	17
135	17	16
95	14	16
92	14	17
60	23	18
69	30	31
90	32	18
82	13	14
139	68	37
61	41	42
388	54	28

141	39	18
115	58	18
270	29	25
85	30	28
399	23	19
333	56	64
ND	79	74
132	50	23
307	29	40
164	25	20
134	38	41
11	36	22
76	20	14
97	21	28
79	25	15

ALT<br>before	ALT<br>after	ALT<br>after-before
20	13	-7
22	20	-2
9	10	1
15	12	-3
10	23	13
11	17	6
13	7	-6
7	7	0
12	11	-1
19	15	-4
15	18	3
12	13	1
15	15	0
14	14	0
18	24	6
16	15	-1
12	12	0
23	20	-3
16	10	-6
12	12	0
19	9	-10
21	22	1
11	11	0
12	9	-3
13	12	-1
13	16	3
11	15	4
21	14	-7
33	38	5
25	14	-11
34	21	-13
55	35	-20
64	66	2
52	41	-11

47	18	-29
76	22	-54
38	18	-20
42	41	-1
40	29	-11
99	123	24
163	86	-77
83	57	-26
48	52	4
32	29	-3
42	31	-11
38	22	-16
26	16	-10
28	27	-1
25	14	-11

Fib-4 index before	Fib-4 index after	Platelet count before
0.86	1.03	44.3
1.74	1.37	26.4
1.46	1.53	27.9
0.66	0.53	26.0
0.29	0.35	46.6
1.14	1.29	23.2
2.02	1.96	18.7
1.18	1.52	27.3
1.07	1.09	22.7
0.70	0.80	27.7
1.81	1.77	20.3
1.13	1.23	31.1
1.20	1.07	22.5
1.20	1.11	21.3
2.37	2.17	17.1
0.95	0.92	29.3
1.62	1.42	25.8
1.85	1.64	18.0
1.34	2.23	19.2
1.55	6.59	15.6
1.24	1.42	29.3
0.15	0.17	23.9
0.11	0.13	18.2
2.65	2.40	17.2
1.63	1.64	22.9
0.91	0.93	32.4
1.54	1.54	23.3
ND	ND	ND
1.67	1.72	26.6
2.47	1.74	19.2
0.37	0.47	32.3
7.11	6.89	12.1
1.71	1.46	24.0
0.88	0.51	31.6

2.70	1.53	14.1
1.59	0.89	23.5
1.63	1.95	19.3
1.14	1.14	22.3
0.41	0.41	30.0
1.72	1.30	14.4
2.28	1.74	19.0
1.73	0.93	18.1
0.71	0.82	28.7
0.79	0.66	30.3
1.09	1.61	24.7
3.21	2.49	14.0
1.33	1.38	20.1
1.34	1.61	21.7
1.96	1.31	19.4

Platelet count after	Hypertension	Dyslipidemia
36.4	0	0
29.7	0	1
25.6	1	1
29.0	0	0
28.2	0	0
21.3	0	0
20.3	1	1
22.9	1	0
25.2	1	0
25.1	1	1
23.0	0	1
30.4	1	1
21.3	1	1
20.6	0	0
17.3	0	0
30.2	1	1
25.9	1	1
19.9	0	1
15.3	0	1
8.4	0	1
30.3	1	1
22.9	1	1
19.1	0	0
19.6	1	1
22.3	1	0
32.7	0	0
24.2	1	0
ND	0	1
25.1	1	1
20.7	1	1
34.4	1	1
8.5	1	1
28.3	0	0
32.5	1	0

18.8	1	0
25.3	0	1
20.5	1	1
21.4	1	1
29.9	0	1
20.0	0	0
32.1	1	0
18.7	0	1
33.2	0	1
30.6	1	1
21.0	1	0
14.7	0	1
17.5	0	1
24.4	1	1
23.2	1	0

Macrovascular diseases	DPP4 inhibitor use before	DPP4 inhibitor use after
0	0	0
0	0	0
0	0	0
0	0	0
0	1	1
0	1	1
0	1	1
0	1	1
0	1	1
0	1	1
1	1	1
1	0	0
0	0	0
0	0	0
0	0	0
0	1	1
1	1	1
0	1	1
0	1	1
0	1	1
0	0	0
0	0	0
0	0	0
0	1	1
0	1	1
0	1	1
0	1	1
0	0	0
1	0	0
0	1	1
0	0	0
0	0	0
0	1	1
0	0	0

0	0	0
0	0	0
1	0	0
1	0	0
0	1	0
0	0	0
0	1	1
0	0	0
0	0	0
0	1	1
1	0	0
0	1	1
0	1	1
0	0	0
0	0	1

SGLT2 inhibitor use before	SGLT2 inhibitor use after	Metformin use before
1	1	0
0	0	1
0	0	1
1	1	1
1	1	0
0	0	0
0	0	1
0	0	1
1	1	1
1	1	0
1	1	0
1	1	1
1	1	1
1	1	0
1	1	0
1	1	1
1	1	1
0	0	0
0	1	1
1	1	0
1	1	0
1	1	0
1	1	0
0	0	0
1	1	1
0	0	1
1	1	0
1	1	1
1	1	0
1	1	1
1	1	1
0	0	1
0	1	0
0	0	1

0	0	0
0	0	0
1	1	0
1	1	0
1	0	1
0	0	1
0	0	0
0	0	1
1	1	1
1	1	1
1	1	1
0	0	0
0	0	1
1	1	1
1	1	1

Metformin use after	Sulfonylurea use before	Sulfonylurea use after
0	0	0
1	1	1
1	0	0
1	1	1
0	0	0
0	0	0
0	0	0
1	1	1
1	0	0
0	1	1
0	0	0
1	0	0
1	0	0
0	0	0
0	0	0
1	1	1
1	1	1
0	1	1
1	0	0
0	0	0
0	0	0
0	0	0
0	0	0
0	0	0
1	0	0
1	1	1
0	1	1
1	1	1
0	1	1
1	1	1
1	1	1
1	0	0
0	0	1
1	1	1

0	0	0
0	0	0
0	0	0
0	0	0
1	0	0
1	0	0
0	1	1
1	0	0
1	0	0
1	0	0
1	0	0
1	1	1
1	0	0
1	0	0
1	0	0

GLP-1RA use before	GLP-1RA use after	GLP-1/GIPRA use before
1	1	0
1	1	0
1	1	0
1	1	0
0	0	0
0	0	0
0	0	0
0	0	0
0	0	0
0	0	0
0	0	0
1	1	0
0	0	0
1	1	0
0	0	0
0	0	0
0	0	0
0	0	0
0	0	0
0	0	0
1	1	0
1	1	0
1	1	0
0	0	0
0	0	0
0	0	0
0	0	0
1	0	0
1	1	0
0	0	0
1	0	0
0	0	0
0	0	0
1	1	0

1	1	0
0	0	0
1	1	0
1	1	0
0	1	0
0	0	0
0	0	0
0	0	0
1	0	0
0	0	0
1	1	0
0	0	0
0	0	0
0	0	0
0	0	0

GLP-1/GIPRA use after	Thiazolidine use before	Thiazolidine use after
0	0	0
0	0	0
0	0	0
0	0	0
0	0	0
0	0	0
0	0	0
0	0	0
0	0	0
0	0	0
0	0	0
0	0	0
0	0	0
0	0	0
0	0	0
0	0	0
0	0	0
0	0	0
0	0	0
0	0	0
0	0	0
0	0	0
0	0	0
0	0	0
0	0	0
0	0	0
0	0	0
1	0	0
0	0	0
0	0	0
1	0	0
0	0	0
0	0	0
0	0	0

0	0	0
0	0	0
0	1	1
0	0	0
0	0	0
0	0	0
0	0	0
0	0	0
1	0	0
0	0	0
0	0	0
0	0	0
0	0	0
0	0	0
0	0	0

Insulin use before	Insulin use after	ACE inhibitor/ARB use before
1	1	0
1	1	0
0	0	0
0	0	0
0	0	0
0	0	0
0	0	0
0	0	1
1	1	0
1	1	0
1	1	0
1	1	1
1	0	1
0	0	0
0	0	0
0	0	0
0	0	1
0	0	0
0	0	0
0	0	0
1	1	0
0	0	1
1	1	1
1	1	0
1	1	1
0	0	0
0	0	1
0	0	1
0	0	1
0	0	1
0	0	1
1	1	0
0	0	0
1	1	1

0	0	1
0	0	0
0	0	1
1	1	1
1	1	0
0	0	0
0	0	0
0	0	0
0	0	0
0	0	1
0	0	1
0	0	0
1	1	0
1	1	1
1	1	1

ACE inhibitor/ARB use after	Statin use before	Statin use after
0	0	0
0	1	1
0	0	0
0	0	0
0	0	0
0	0	0
0	1	1
1	0	0
0	0	0
0	1	1
0	1	1
1	1	1
1	0	0
0	0	0
0	1	1
0	0	0
1	1	1
0	1	1
0	1	1
0	1	1
0	0	0
1	0	0
1	0	0
0	1	1
1	1	1
0	0	0
1	0	0
1	0	0
1	1	1
1	1	1
1	1	1
0	1	1
0	0	0
1	0	0

1	0	0
0	0	0
1	0	0
1	1	1
0	0	0
0	0	0
0	0	0
0	0	0
0	0	0
1	1	1
1	0	0
0	1	1
0	1	1
1	1	1
1	0	0

Fibrate use before	Fibrate use after	Pemafibrate use before
0	0	0
0	0	0
0	0	0
0	0	0
0	0	0
0	0	0
0	0	0
0	0	0
0	0	0
0	0	0
0	0	0
0	0	1
0	0	0
0	0	0
0	0	0
0	0	0
0	0	0
0	0	0
0	0	0
0	0	0
1	1	0
0	0	0
0	0	0
0	0	0
0	0	0
0	0	0
0	0	0
1	1	0
0	0	0
0	0	0
0	0	1
0	0	0
0	0	0
0	0	1

0	0	0
0	0	0
0	0	0
0	0	0
0	0	0
0	0	0
0	0	0
0	0	0
0	0	0
0	0	0
1	1	0
0	0	0
0	0	0
0	0	0
0	0	0

Pemafibrate use after	Antiplatelet use before	Antiplatelet use after
0	0	0
0	0	0
0	0	0
0	0	0
0	0	0
0	0	0
0	0	0
0	0	0
0	0	0
0	0	0
0	0	0
1	1	1
0	0	0
0	0	0
0	0	0
0	0	0
0	1	1
0	0	0
0	0	0
0	0	0
0	0	0
0	0	0
0	0	0
0	1	1
0	0	0
0	0	0
0	0	0
0	0	0
0	1	1
0	0	0
1	0	0
0	1	1
0	0	0
1	0	0

0	0	0
0	0	0
0	1	1
0	1	1
0	0	0
0	0	0
0	0	0
0	0	0
0	0	0
0	0	0
0	0	0
0	0	0
0	0	0
0	0	0
0	0	0

Initiated/Discontinued SGLT2i, GLP-1RA, GLP-1/GIPRA
0
0
0
0
0
0
0
0
0
0
0
0
0
0
0
0
0
0
1
0
0
1
1
0
0
0
0
1
0
0
1
0
1
0

0
0
0
0
1
0
0
0
1
0
0
0
0
0
0
